# The Comparison of Teen Clubs vs. Standard Care on Treatment Outcomes for Adolescents on Antiretroviral Therapy in Windhoek, Namibia

**DOI:** 10.1155/2020/8604276

**Published:** 2020-10-27

**Authors:** Farai K. Munyayi, Brian E. van Wyk

**Affiliations:** University of the Western Cape, Cape Town, South Africa

## Abstract

**Background:**

Adolescents living with HIV (ALHIV) are challenged to adhere to antiretroviral therapy (ART) and achieve and maintain virologic suppression. Group-based adherence support interventions, such as adherence clubs, have been shown to improve long-term adherence in ART patients. The teen club intervention was introduced in 2010 in Namibia to improve treatment outcomes for ALHIV by providing adherence support in a peer-group environment. Adolescents who have completed the full HIV disclosure process can voluntarily join the teen clubs. The current study compared treatment outcomes of ALHIV receiving ART at a specialized paediatric HIV clinic between 1 July 2015 and 30 June 2017 in Windhoek, Namibia.

**Methods:**

A retrospective cohort analysis was conducted on routine patient data extracted from the electronic Patient Monitoring System, individual Patient Care Booklets, and teen club attendance registers. A sample of 385 adolescents were analysed: 78 in teen clubs and 307 in standard care. Virologic suppression was determined at 6, 12, and 18 months from study start date, and compared by model of care, age, sex, disclosure status, and ART regimen. Comparisons between adolescents in teen clubs and those receiving standard care were performed using the chi-square test, and risk ratios were calculated to analyze differences in ART adherence and virologic suppression.

**Results:**

The average clinician-measured ART adherence was 89% good, 6% fair, and 5% poor amongst all adolescents, with no difference between teen club members and adolescents in standard care (*p* = 0.277) at 3 months. Virologic suppression over the 2-year observation period was 87% (68% fully suppressed <40 copies/ml and 19% suppressed between 40–999 copies/ml), with no difference between teen club members and those in standard care. However, there were statistically significant differences in virologic suppression levels between the younger (10–14 years) adolescents and older (15–19 years) adolescents at 6 months (*p* = 0.015) and at 12 months (*p* = 0.021) and between adolescents on first-line and second-line ART regimen at 6 months (*p* = 0.012), 12 months (*p* = 0.004), and 18 months (*p* = 0.005).

**Conclusion:**

The teen club model delivering psychosocial support only did not improve adherence and virologic suppression levels for adolescents in a specialized paediatric ART clinic, neither were they inferior to standard care. Considering the limitations of this study, teen clubs may still hold potential for improving adherence and virologic suppression levels for older adolescents, and more robust research on adherence interventions for adolescents with higher methodological quality is required.

## 1. Introduction

Due to the successes in prevention of mother-to-child transmission (PMTCT) programs worldwide and advances in paediatric HIV treatment, children with HIV are surviving to reach adolescence [1]. The World Health Organization defines adolescents as children or young adults between 10 and 19 years of age [2]. Worldwide in 2018, an estimated 1.6 million adolescents between 10 and 19 years were living with HIV, with nearly 85% living in sub-Saharan Africa [3, 4]. In most sub-Saharan Africa countries, public health facilities are ill-equipped to give guidance and support for adolescents living with HIV (ALHIV) to remain engaged in care and adhere to medication regimens [5]. In 2019 alone, there were 460,000 newly infected young people between the ages of 10 to 24 years, of whom 170,000 were adolescents between 10 to 19 years [6].

Poor ART adherence increases the risk of viral drug resistance, limits treatment efficacy, leading to disease progression, and reduces future therapeutic options as well as increasing the risk of transmission due to unsuppressed viral replication [7]. Although reported ART adherence is high globally (>95%), concerns have been raised about waning adherence over time including loss of patients from HIV programs when scaling up [8]. Evidence-based interventions to address adherence challenges for people on ART include individual and group adherence counselling, mHealth platforms, community and home-based strategies, pharmacist counselling and monitoring, task-shifting, medication fast-tracking, nutrition support, and provision of disability grants [9]. For ALHIV, individual counselling, group counselling, and peer support, such as in teen clubs, have been some of the most common interventions in Namibia.

Namibia has adopted the Joint United Nations Programme on HIV and AIDS' (UNAIDS) fast track goals to achieve HIV epidemic control by 2030. The fast track goals are aimed at ensuring that 95% of PLHIV are identified; 95% of those identified are effectively linked and retained on ART; and 95% of these achieve virologic suppression [10]. Adolescents living with HIV have unique needs and are notably underserved globally and in national responses, which negatively affects their access to ART and results in poor ART adherence and inferior treatment outcomes such as achieving and maintaining virologic suppression [11]. In Namibia, infants, children, and younger adolescents (0–14 years) reportedly had only 63% viral load suppression, and young people (older adolescents and young adults, 15–24 years old) had 60.5%, which is well below the national average suppression levels for adults on ART at 80.5% [12].

According to WHO, a maintained viral load of <1000 ribonucleic acid (RNA) copies per ml of plasma is considered evident of virologic suppression [13]. According to the 2019 Namibia National Guidelines for Antiretroviral Treatment, virologic status is classified into three categories, namely, fully suppressed (<40 copies/ml), suppressed (40–999 copies/ml), and unsuppressed (≥1000 copies/ml). The aim of this classification is for earlier identification of patients having suboptimal responses to therapy, whose immunologic and clinical responses may not have deteriorated at this stage, but persistently have viral loads of above 40 copies/ml. These patients undergo different clinical management, which includes intensive adherence counselling and support to achieve full suppression and avoid treatment failure that may necessitate switching to a second-line ART regimen [14].

A teen club intervention was established in 2010 at a paediatric HIV clinic, in Windhoek, to address unique needs of adolescents on HIV treatment [15]. The teen club aims to improve ART adherence through, among other activities, psychosocial support, HIV counselling, and health education. In 2010, teen club interventions were introduced at health facilities in Malawi to provide ALHIV on ART with dedicated clinic time, peer mentorship, sexual and reproductive health education, ART refill and support for positive living, and treatment adherence. An evaluation of the program in 2015 found that ALHIV with no teen club exposure were less likely to be retained than those with teen club exposure (adjusted odds ratio (aOR) 0.27; 95% CI 0.16, 0.45). ALHIV aged 15–19 years were more likely to have attrition from care than those aged 10–14 years (aOR 2.14; 95% CI 1.12, 4.11) [16]. Another evaluation in Malawi of a similar teen club intervention reported in 2019 on adherence levels between younger and older adolescents and male and female adolescents found that older adolescence were associated with higher odds of optimal adherence compared to younger adolescents (aOR 1.48; 95% CI 1.16–1.90, *p* < 0.01) [17]. Evaluations of teen clubs have been scarce, and both Malawi studies recommended age-specialized programming for adolescents and argued that more prospective research is required with higher methodological quality.

To date, the effectiveness of the teen clubs on adolescents' ART adherence has not been formally evaluated in Namibia. This paper reports on the effects of the teen club intervention against standard care on ART adherence and virologic suppression amongst adolescents at the clinic.  Table 1 shows services provided in standard care compared to the teen club. The main difference between standard care and the teen club is that the teen club provides a group-based psychosocial support platform, which meets outside of the routine clinic visits schedule to share experiences, deliver presentations, engage in educational activities, to keep the adolescents engaged in care and on ART, and improve their overall we-being.

## 2. Methods

### 2.1. Study Setting, Design, and Population

A retrospective cohort study was conducted using medical records of HIV positive adolescents between ages of 10 and 19 years receiving ART at a hospital-based paediatric HIV clinic in Windhoek, Namibia. The paediatric ART clinic is a specialized HIV clinic with dedicated staff for paediatric HIV management, which includes a physician, nurses, counsellors, and other support staff. The study population was stratified into two groups of adolescents attending the teen club and adolescents who were receiving standard care. Routine clinical records of the study population from 1 July 2015 to 30 June 2017 were reviewed. All adolescents between the ages of 10 and 19 years attending the clinic between 1 July 2015 and 30 June 2017 as their initial enrolment site were eligible for inclusion in the study. There were no changes in the treatment guidelines or clinical intervention during the study period.

### 2.2. Participants' Selection

**Figure 1:**
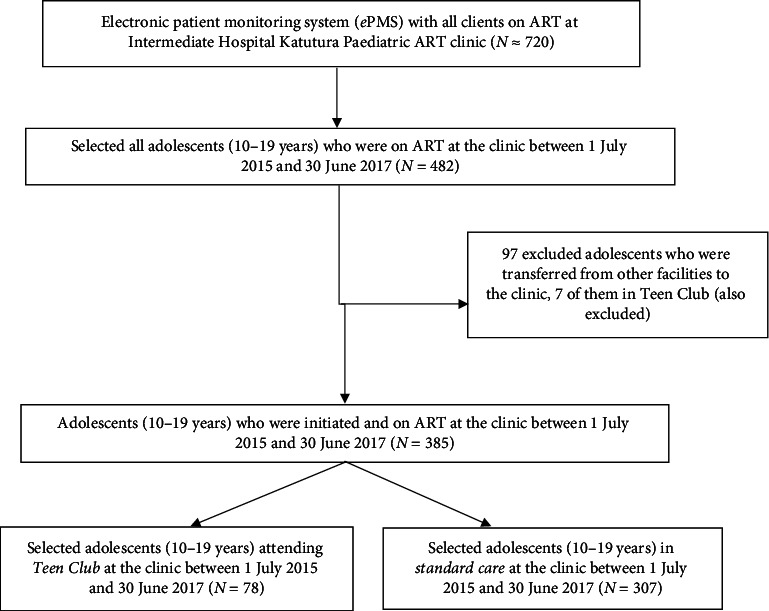
Flow chart of sampling process for the study.

The study sample was all inclusive of the study population.  Figure 1 below shows that an estimated 720 children and adolescents were receiving ART at the clinic and 482 of them were aged between 10 and 19 years with 85 being members of the teen club. According to the 2014 Namibian National ART Guidelines, the child HIV disclosure process should be initiated as early as 6 years to 10 years of age [18]. Once adolescents are aware that they are HIV infected, they become eligible to enroll in the teen club, and a clinician can facilitate enrolment of the child into the teen club, in consultation with the caregiver of the child. To standardize exposure to the service delivery model at the specialized clinic, all adolescents transferred in from other clinics were excluded from the study, including 7 in the teen club. Any adolescent who attended at least one teen club meeting was considered exposed to the intervention. Sixteen [16] teen club meetings were held during the study period with an average of 42 adolescents attending each meeting and each adolescent attending an average of 5 meetings over the 2-year period.

The calculated minimum total sample size using Epi-Info was 272 participants, with 46 from the teen club stratum (exposed) and 226 in the standard care stratum (unexposed). Parameters used to calculate sample size include a power of 80%, an assumed 20% difference between the two groups, with a 95% confidence interval, and an unexposed/exposed ratio of 5.

### 2.3. Data Abstraction and Management

Patient demographics and visit details are completed routinely by healthcare workers into individual Patient Care Booklets (PCBs) during clinic visits. Patient information is then entered into an electronic Patient Monitoring System (*e*PMS) by data clerks. Patient data was extracted from the electronic database into an Excel spreadsheet. Teen club members sign in at every teen club meeting. The teen club register was reviewed to match adolescents on the *e*PMS and the teen club members using the unique ART numbers allocated as unique patient identifiers.

Patient Care Booklets for adolescents with incomplete records in *e*PMS were retrieved, and the missing information was added to the Excel spreadsheet. Extracted data was saved onto a password protected Excel file to ensure that the data could not be altered. Data cleaning and preparation/coding were done on the Excel file which was then exported into a Stata16 file for further processing and analysis.

### 2.4. Variables

Adherence to ART was assessed through patient self-reports and pill counts conducted by clinicians during the 24-month study period. Clinicians ask the patients about the number of missed doses per month and conduct a physical pill-count. Virologic suppression has been used in several ART adherence studies as the standard biomarker of adherence levels and evidence shows that it is a reliable predictor of good adherence [19]. Once viral load results are received from the laboratory, they are recorded in the PCBs and subsequently entered on the *e*PMS. Missing viral load results on the *e*PMS were extracted from the PCBs. The most important exposure variable assessed is the model of care, with teen club members in the exposed group and adolescents in standard care in the unexposed group. Other predictor variables of interest that were collected included demographic and clinical characteristics such as age, sex, period on ART, current ART regimen type, and HIV disclosure status. While this paper focuses on viral load suppression, measured at three data collection points from the study start date (months 6, 12, and 18), we also analysed documented adherence at 3 monthly intervals from study start date and overall retention in care (attending clinic visits) at 24 months from study start date, which has been reported elsewhere [20].

### 2.5. Statistical Analyses

The dataset in Excel was exported to Stata for the data analysis, and the analysis was done using the Stata statistical software, release 16, College Station, TX, StataCorp LLC. The data analysis included univariate analyses to describe demographic variables such as sex and age distribution; clinical variables such as model of care, disclosure status, period on ART, and type of regimen; and virologic suppression and adherence measured by clinicians. Bivariate analysis was performed using the chi-square test to determine the significance of associations between ART adherence and virologic suppression and selected demographic and clinical variables. Cut-off for significance of associations was set at *p* < 0.05. If the sample size was very small in any cell (<5), Fisher's exact test was used as an alternative to the chi-square test. Relative risk was also calculated for comparison of ART adherence and virologic suppression levels between teen club members and adolescents in standard care. Calculation of relative risk was conducted using the Poisson regression with the *glm* command in Stata. Multivariate modeling was also performed to determine the relative risk adjusting for age and sex, disclosure, and type of ART regimen.

This study was approved by the Namibian Biomedical Research Ethics Committee and Research Management Committee based at the Ministry of Health and Social Services (Ref: 18/3/3 FM) and the University of the Western Cape Biomedical Research Ethics Committee (Ref: BM17/8/14).

## 3. Results

A total of 482 adolescents aged between 10 and 19 years attended the paediatric ART clinic during the 2-year study period. All records of the adolescents were extracted from the *e*PMS for the two-year period. Records of adolescents who were transferred in from other facilities, who had incorrectly entered demographic information, and who are with missing files/PCBs were excluded from the final study sample. A total 385 adolescents were eligible, 78 of them being in the teen club. The average age amongst adolescents included in the study was 14 years and 51% were aged 10–14 years whilst 49% were aged 15–19 years. However, as presented in Table 2, the proportion of older adolescents in the teen club was much higher than that of the older adolescents in standard care (66.7% vs. 44.3%; *p* = 0.015), and the same is true for the female adolescents enrolled in the teen club compared to standard care (59% vs. 43.6%; *p* = 0.001). The average measured ART adherence by the clinicians during clinical visits was 89% good (≥95%), 6% fair (85–94%), and 5% poor (<85%) amongst all the adolescents over the 2 years.

All the adolescents in the teen club had their HIV status disclosed to them (a requirement for enrolment in the club), compared to 94% of adolescents in standard care (*p* = 0.031) (Table 1). The median duration on ART among all participating adolescents was 10.3 years (interquartile range = 7.7–11.7), mean duration on ART of 9.4 years, with a minimum of 1 month and a maximum of 17.4 years on ART. There was no significant difference between teen club members and those in standard care on duration on ART (100% vs. 99% 12 months or more), type of ART regimen (68% vs. 74% on first line), adherence at 3 months (95% vs. 90% good adherence), and retention status at 24 months (91% vs. 90% retained in care).

**Figure 2:**
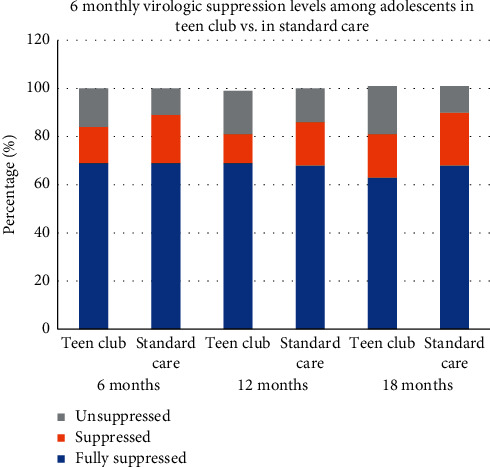
Virologic suppression levels among adolescents on antiretroviral therapy by model of care.

Viral load suppression amongst the adolescents who were included in the study at the Paediatric ART clinic was on average at 87% (68% fully suppressed and 19% suppressed) and 13% not suppressed for the 2-year study period. Full suppression is defined as a viral load <40 copies/ml or Target not Detected (TND), whilst individuals with a viral load between 40 and 999 copies/ml are categorized as suppressed.  Figure 2 shows that a similar trend was observed during the 2-year period, at 6-month, 12-month, and 18-month clinic visits during the study period.

Table 3 shows that there was no sufficient evidence of a statistically significant difference in viral load suppression levels at 6 months, 12 months, and 18 months between teen club members and adolescents in standard care. No statistically significant differences were observed in viral load suppression levels between adolescents whose HIV status was disclosed to and those who were not disclosed to at 5% significance level. There was sufficient evidence of statistically significant differences in viral load suppression at 5% significance level between the younger (10–14 years) adolescents and older (15–19 years) adolescents at 6 months and at 12 months, but there was no statistically significant difference at 18 months at 5% level. There was also sufficient evidence of a statistically significant difference in viral load suppression between adolescents on a first line ART regimen and those on second line at 6 months, at 12 months, and at 18 months. It is logical that the adolescents on first-line ART regimens were more likely to be suppressed than those on a second-line regimen since transition to a second-line regimen may be indicative of treatment failure.

Table 4 shows that, at 6 months' and 12 months' follow-up, the adjusted relative risk was significant for age and the type of ART regimen, with younger adolescents and those on a first-line regimen having better viral load suppression levels than the older adolescents and adolescents on a second-line ART regimen, respectively. There was some evidence that older adolescents may be less likely to achieve viral suppression at 6, 12, and 18 months although this effect largely disappeared after adjusting for sex, disclosure, and type regimen. There were no significant differences observed according to sex or disclosure status of the adolescents.

At 18 months, the adjusted relative risk for all variables showed no significant differences. After controlling for age, sex, disclosure status, and type of ART regimen, the model of care did not significantly influence viral load suppression at 6 months (*p* = 0.927), 12 months (*p* = 0.324), and 18 months (*p* = 0.506). Compared to the primary findings, our study found no significant differences in the sensitivity analysis on viral load suppression rates between teen club members and adolescents in standard care by age groups at 6 months (*p* = 0.819) and 12 months (*p* = 0.427).

## 4. Discussion

The overall viral load suppression rates among the adolescents included in the study were at 87%—which almost reached the initial UNAIDS target of 90%. Considering that this study also found the overall retention in care rates to be approximately 90.1% at 24 months among all adolescents included in the study, it is indeed encouraging as it holds hope for achieving the 95-95-95 targets. Similar viral load suppression levels among adolescents have been reported in other studies in Africa and in the sub-Saharan Africa region. In South Africa, viral load suppression rates were reported to be 81% among adolescents using a cut-off of <400 copies/ml to define HIV-1 viral suppression [21]. A literature review conducted in 2016 showed wider variations in studies that assessed viral load suppression rates among adolescents at 12 months after ART initiation, with viral load suppression rates ranging from 27% to 89% in studies stratified by duration on ART and 28% to 87% in studies not stratified by duration on ART [22]. This points to a greater need for adolescent-friendly HIV services in national programs to breach the 90% virologic suppression goal initially set by global programs.

This study did not show any significant differences in viral load suppression rates between teen club members and adolescents who were receiving standard care. Overall, the viral load suppression rates were relatively high among all adolescents included in the study. Considering that the study setting is a specialized paediatric HIV clinic, the quality of HIV care and treatment outcomes would generally be expected to be better than HIV care in an integrated primary healthcare (PHC) facility where you may not have dedicated specialized staff to manage paediatric HIV patients. The 2015 Namibia Preliminary Report for Adolescents Assessment reported viral load suppression rates of 74% and 70% among adolescent girls and boys, respectively, and 73% and 63% among 10–14 year and 15-19 year olds, respectively [14]. Our study population consists mostly of adolescents who had been receiving specialized HIV care for more than 12 months, with a median period on ART of 10.3 years. This points to mostly treatment-experienced adolescents, and it is expected that generally viral load should reach undetectable levels by 6 months of therapy in fully adherent clients [14]. The meta-analysis done by Ferrand et al. reported on varied published viral load suppression results among adolescents and emphasized the need to report suppression rates stratified by duration on ART and reporting of median duration on ART so as to contextualize treatment outcomes [22].

Another study in South Africa showed that there were better viral suppression levels in an adolescent-friendly HIV clinic (91%) compared to those in a standard paediatric clinic (80%) [21]. However, the adolescent-friendly clinic was based on a differentiated care model, providing HIV medication, psychosocial and peer support, education and sports, lunches, and other entertainment activities through a Saturday clinic [21]. The model for the adolescent-friendly clinic reduced school absenteeism and stigma in addition to the services provided, resulting in better viral suppression levels and retention in care. Our study setting already provided a high level of standard of care, with the teen club only as an additional psychosocial support intervention, which may have resulted in a limited impact of the teen club intervention.

Our results suggest that the teen club intervention model implemented at the specialized paediatric HIV clinic may not offer adequate additional benefits to the club members on viral suppression, which may depend on other factors besides levels of adherence to treatment. Several reports have also showed reduced viral suppression levels amongst older adolescents and considering that most of the teen club members in our study were older adolescents, age could have influenced viral suppression levels among club members. The results showed that the teen club consists of twice as many older adolescents compared to younger adolescents and that the teen club had a higher proportion of older adolescents as compared to the standard care arm.

There was a significant difference in viral load suppression levels between older and younger adolescents, with the older adolescents having less chance of viral load suppression compared to younger adolescents, although this difference was minimal and reduced after adjusting for sex, disclosure, and ART regimen. Similar results have been described in other studies in Africa, with Evans et al. reporting that older adolescents were more likely to be unsuppressed as compared to their younger counterparts at 6 months in a study conducted in adolescents and young adults in Mpumalanga (RR = 1.75; 95% CI = 1.25–2.47) [23]. The association between age and viral suppression was also described as a significant factor in other studies that compared viral suppression levels among young adults and adolescents, showing that younger adolescents had higher rates of viral load suppression [19, 20]. As discussed earlier, the paediatric HIV clinics provide a more favourable environment for younger adolescents in general, and the younger adolescent is more likely to have better treatment outcomes due to closer caregiver support. The Windhoek teen club intervention model may need to strengthen the efforts already underway to deliver content developmentally tailored by age group.

Overall, the standard of care at the specialized paediatric HIV clinic was already high with high viral load suppression rates than reported elsewhere in Namibia. Indeed, as reported elsewhere many HIV programs in low- and middle-income countries may not have very strong standard of care models for adolescents to achieve similar treatment outcomes as the specialized paediatric clinic, as such the results may not be generalizable. Therefore, more research on the impact of group-based interventions such as teen clubs in settings where there are no specialized services is needed.

## 5. Conclusions

The teen club model delivering psychosocial support only did not improve adherence and virologic suppression levels for adolescents in a specialized paediatric ART clinic, neither were they inferior to standard care. Considering the limitations of this study, teen clubs may still hold potential for improving adherence and virologic suppression levels for older adolescents, and more robust research on adherence interventions for adolescents with higher methodological quality is required.

## 6. Limitations to the Study

The use of a retrospective cohort study design meant that we had to rely on the accuracy of the record keeping by the healthcare workers. Reliance on routinely collected data meant that we could not control the exposures and outcomes of interest and could not account for unmeasured factors due to the limitations of routinely collected variables. The amount of missing data and files could have potentially influenced the findings of the results. The inequivalence in sample sizes between the comparison groups (teen club members vs. adolescents in standard care) could also potentially result in a type II error. Another limitation is the lack of detailed contents of each teen club session but just an overall description of topics discussed in general. This information could put some of the results into context and facilitate discussions into modifying the intervention where necessary. The dose effect was also not thoroughly investigated, as a single attendance to the teen club was considered as exposure to the intervention.

## Figures and Tables

**Table 1:** Comparison of standard care and teen club care.

Model of care
Similarities between teen club and standard care
3 monthly clinical visits except in high viral load patients who may be enrolled in monthly adherence counselling
Adolescents should have full disclosure by age 10–12; disclosure can be delayed depending on the cognitive ability of the adolescent
Goal-related transition from paediatric/adolescent to adult HIV services
Routine viral load monitoring and targeted viral load monitoring for suspected treatment failure
Age-appropriate and developmentally appropriate adherence counselling
Lost to follow-up/defaulter tracking and tracing
HIV treatment literacy training of guardians and caregivers on treatment adherence, disclosure, and stigma issues
Age-appropriate psychosocial support includes individualized counselling on issues such as treatment failure counselling, opportunistic infections, STIs, sexual and reproductive health, alcohol use and abuse, mental health, child protection, and other topics according to the adolescents' needs
Routine discussion with the child on their experience at school and future plans
Linkage to relevant stakeholders and social support mechanisms in the community
Additional considerations and support in teen clubs
Adolescents should have full disclosure; this is a prerequisite for enrolment into the teen club; adolescents can enroll once disclosed to
In addition to age-appropriate psychosocial support offered in standard care, the teen club
Meets once a month on a Friday or Saturday in “safe spaces” at the clinic
Share challenges, fears, experiences, and coping mechanisms during monthly meetings
Have special talks or presentation of ALHIV-related topics from subject matter experts
Have access to information, education, and communication materials such as videos and dramas/acts on adolescence and HIV and have discussions thereafter
Occasionally participate in teen club retreats and trips where recreational activities and life stories are shared

**Table 2:** Demographic and clinical characteristics of adolescent participants on ART at Intermediate Hospital Katutura Paediatric ART Clinic (*N* = 385).

Characteristic	Total	Standard care (%)	Teen club (%)	*p* value
Sex				
Male	205	173 (56.4)	32 (41.0)	0.015^*∗*^
Female	180	134 (43.6)	46 (59.0)	
Age group				
10–14 years	197	171 (55.7)	26 (33.3)	0.001^*∗∗*^
15–19 years	188	136 (44.3)	52 (66.7)	
Disclosure status (*n* = 372)				
Disclosed	355	278 (94.2)	77 (100)	0.031^*∗*^
Not disclosed	17	17 (5.8)	0 (0)	
ART regimen				
First-line regimen	279	226 (73.6)	53 (67.9)	0.318
Second-line regimen	106	81 (26.4)	25 (32.1)	
Duration on ART				
<12 months	3	3 (1.0)	0 (0)	0.382
≥12 months	382	304 (99.0)	78 (100)	
Adherence at 3 months				
Good	350	276 (90)	74 (95)	0.277
Fair	18	17 (6)	1 (1)	
Poor	15	3 (4)	12 (4)	
Retention status at 24 months				
In care	347	276 (89.9)	71 (91.0)	0.931
Lost to follow-up	22	18 (5.9)	4 (5.1)	
Transfer out	16	13 (4.2)	3 (3.9)	

^*∗*^Correlation is significant at 0.05 level (2-tailed). ^*∗∗*^Correlation is significant at 0.01 level (2-tailed).

**Table 3:** Virologic suppression by demographic and clinical characteristics.

Characteristics	6 months			*p* value	12 months			*p* value	18 months			*p* value
	Fully suppressed	Suppressed	Unsuppressed		Fully suppressed	Suppressed	Unsuppressed		Fully suppressed	Suppressed	Unsuppressed	
Model of care												
Teen club	51 (69%)	11 (15%)	12 (16%)	0.298	50 (69%)	9 (12%)	13 (18%)	0.438	44 (63%)	12 (17%)	14 (20%)	0.113
Standard care	195 (69%)	57 (20%)	30 (11%)		192 (68%)	50 (18%)	39 (14%)		175 (68%)	56 (22%)	28 (11%)	

Age group												
10–14 years	134 (72%)	38 (21%)	13 (7%)	0.015^*∗*^	135 (73%)	31 (17%)	18 (10%)	0.021^*∗*^	122 (71%)	33 (19%)	16 (9%)	0.091
15–19 years	112 (65%)	30 (18%)	29 (17%)		107 (63%)	28 (17%)	34 (20%)		97 (61%)	35 (22%)	26 (16%)	

Sex												
Male	126 (68%)	38 (20%)	22 (12%)	0.793	130 (69%)	35 (19%)	24 (13%)	0.38	113 (64%)	38 (22%)	25 (14%)	0.581
Female	120 (71%)	30 (18%)	20 (12%)		112 (68%)	24 (15%)	28 (17%)		106 (69%)	30 (20%)	17 (11%)	

Disclosure												
Disclosed	233 (70%)	63 (19%)	39 (12%)	0.614	230 (69%)	57 (17%)	47 (14%)	0.122	207 (66%)	67 (21%)	39 (12%)	0.325
Not disclosed	11 (69%)	2 (12%)	3 (19%)		10 (62%)	1 (6%)	5 (31%)		11 (73%)	1 (7%)	3 (20%)	

ART regimen												
First-line	192 (74%)	43 (17%)	23 (9%)	0.012^*∗*^	189 (74%)	36 (14%)	32 (12%)	0.004^*∗*^	168 (71%)	47 (20%)	22 (9%)	0.005^*∗*^
Second-line	54 (55%)	25 (26%)	19 (19%)		53 (55%)	23 (24%)	20 (21%)		51 (55%)	21 (23%)	20 (22%)	

^*∗*^indicates statistical significance at 5% level.

**Table 4:** Relative risk regression for determinants of viral suppression.

Characteristic	6 months		12 months		18 months	
	Crude RR (95% CI)	Adjusted RR (95% CI)	Crude RR (95% CI)	Adjusted RR (95% CI)	Crude RR (95% CI)	Adjusted RR (95% CI)
Model of care						
Teen club	**0.94** (0.84–1.05)	**0.99** (0.89–1.16)	**0.95** (0.85–1.07)	**1.06** (0.94–1.20)	**0.90** (0.79–1.02)	**0.96** (0.85–1.08)
Standard care	1.00	1.00	1.00	1.00	1.00	1.00

Age group						
15–19 years	**0.89** (0.83–0.97)^*∗*^	**0.98** (0.97–0.99)^*∗*^	**0.89** (0.81–0.97)^*∗*^	**0.96** (0.94–0.98)^*∗*^	**0.92** (0.85–1.00)	**0.98** (0.95–1.00)
10–14 years	1.00	1.00	1.00	1.00	1.00	1.00

Sex						
Male	**0.99** (0.93–1.08)	**1.03** (0.97–1.10)	**1.05** (0.96–1.15)	**1.02** (0.94–1.10)	**0.97** (0.89–1.05)	**0.99** (0.93–1.06)
Female	1.00	1.00	1.00	1.00	1.00	1.00

Disclosure						
Not disclosed	**1.09** (0.86–1.38)	**1.11** (0.88–1.40)	**1.25** (0.90–1.74)	**1.38** (0.98–1.94)	**1.09** (0.85–1.41)	**1.18** (0.92–1.53)
Disclosed	1.00	1.00	1.00	1.00	1.00	1.00

ART regimen						
Second	**1.13** (1.02–1.25)^*∗*^	**1.13** (1.02–1.25)^*∗*^	**1.11** (0.99–1.24)	**1.10** (1.00–1.22)^*∗*^	**1.16** (1.03–1.30)^*∗*^	**1.12** (0.65–1.49)
First	1.00	1.00	1.00	1.00		1.00

The bold values are the actual relative risk (RR) figures, and ^*∗*^indicates RR and aRR values that were significant at 5% level.

## Data Availability

The data used to support the findings of this study are available within the article.
